# Author Correction: A mechanical ratchet drives unilateral cytokinesis

**DOI:** 10.1038/s41586-026-10618-0

**Published:** 2026-05-12

**Authors:** Alison Kickuth, Urša Uršič, Michael F. Staddon, Jan Brugués

**Affiliations:** 1 Cluster of Excellence Physics of Life, Technische Universität Dresden, Dresden, Germany; 2 Max Planck Institute of Molecular Cell Biology and Genetics, Dresden, Germany; 3 Max Planck Institute for the Physics of Complex Systems, Dresden, Germany; 4 Center for Systems Biology Dresden, Dresden, Germany

**Keywords:** Microtubules, Biophysics, Cytokinesis, Embryology

Correction to: *Nature* 10.1038/s41586-025-09915-x Published online 7 January 2026

In the version of the article initially published, the Fig. 3h *y*-axis was “G′ (Pa), G″ (Pa s)” but should have been “G′ (Pa), G″ (Pa)”. This correction has been made to the HTML and PDF versions of the article.

