# Supplementary material for: A mechanical ratchet drives unilateral cytokinesis
Source: Nature. 2026 Jan 7;650(8102):759–67. doi: 10.1038/s41586-025-09915-x (PMC12916326; doi:10.1038/s41586-025-09915-x)
Supplement: Supplementary file 1 — This file contains Supplementary Figs. 1–6 and Supplementary Tables 1 and 2. [file 41586_2025_9915_MOESM1_ESM.pdf]

nature portfolio

https://doi.org/10.1038/s41586-025-09915-x

Supplementary information

A mechanical ratchet drives unilateral cytokinesis

In the format provided by the authors and unedited

Supplementary figures and legends

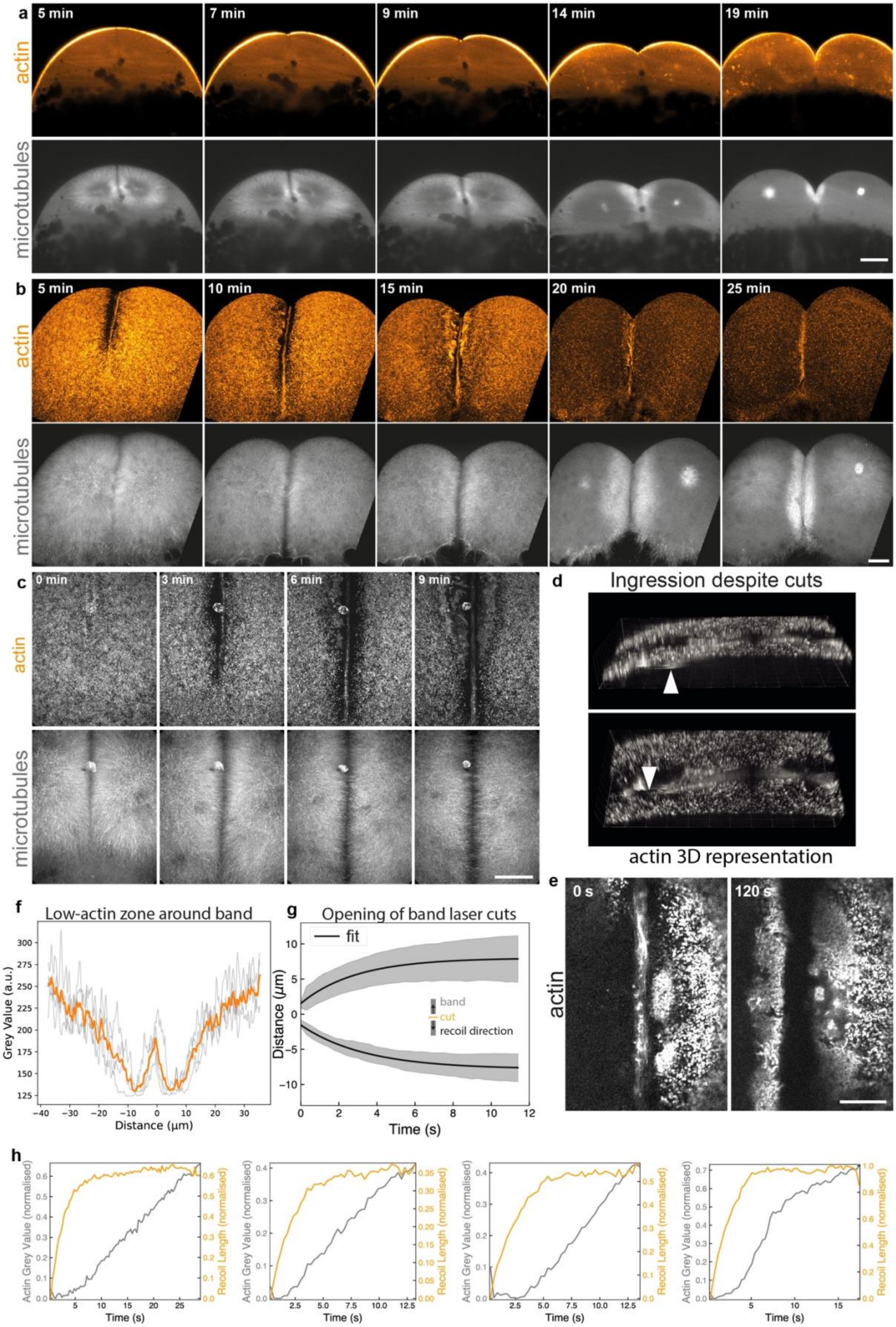

Figure S1

a, Light sheet image of furrow formation and ingression in an unconfined embryo (imaged through chorion), single z-plane within the embryo. Scale bar, 100 µm. **b,** Maximum intensity projection of embryo labelled for actin and microtubules, shown from side, visualising the band end. Scale bar, 50 µm. **c,** Maximum intensity projection of embryo labelled for actin and microtubules visualising the band formation at high resolution, single channel images, related to figure 1b. Scale bar, 50 µm. **d,** 3D visualisations of contractile band after continuous laser cutting. White arrows indicate ingressed band. **e,** Single confocal imaging plane of the contractile band without perturbations, control for figure 1d. Scale bar, 20 µm. **f,** Actin intensity (grey value) across the contractile band from a perpendicular line profile. Actin intensity decreases on either side of the band (centre), measured in N=4 embryos, orange line shows mean, grey lines show individual measurements. **g,** Quantification of laser cuts during the first cell division. Opening of the cut tracked at newly created ends, measured band in N=6 embryos. Black line shows exponential fit $y = a \cdot \left(1 - e^{-\frac{x-c}{b}}\right)$ shaded region shows standard deviation. a= -7.83±0.04, b= 3.39±0.07 and a=8.00±0.02, b=2.89±0.04 for the two recoil directions, respectively. **h,** individual examples (N=1 each) of recoil length following laser cut, compared to actin grey value as measure for healing of the band, related to Fig1e.

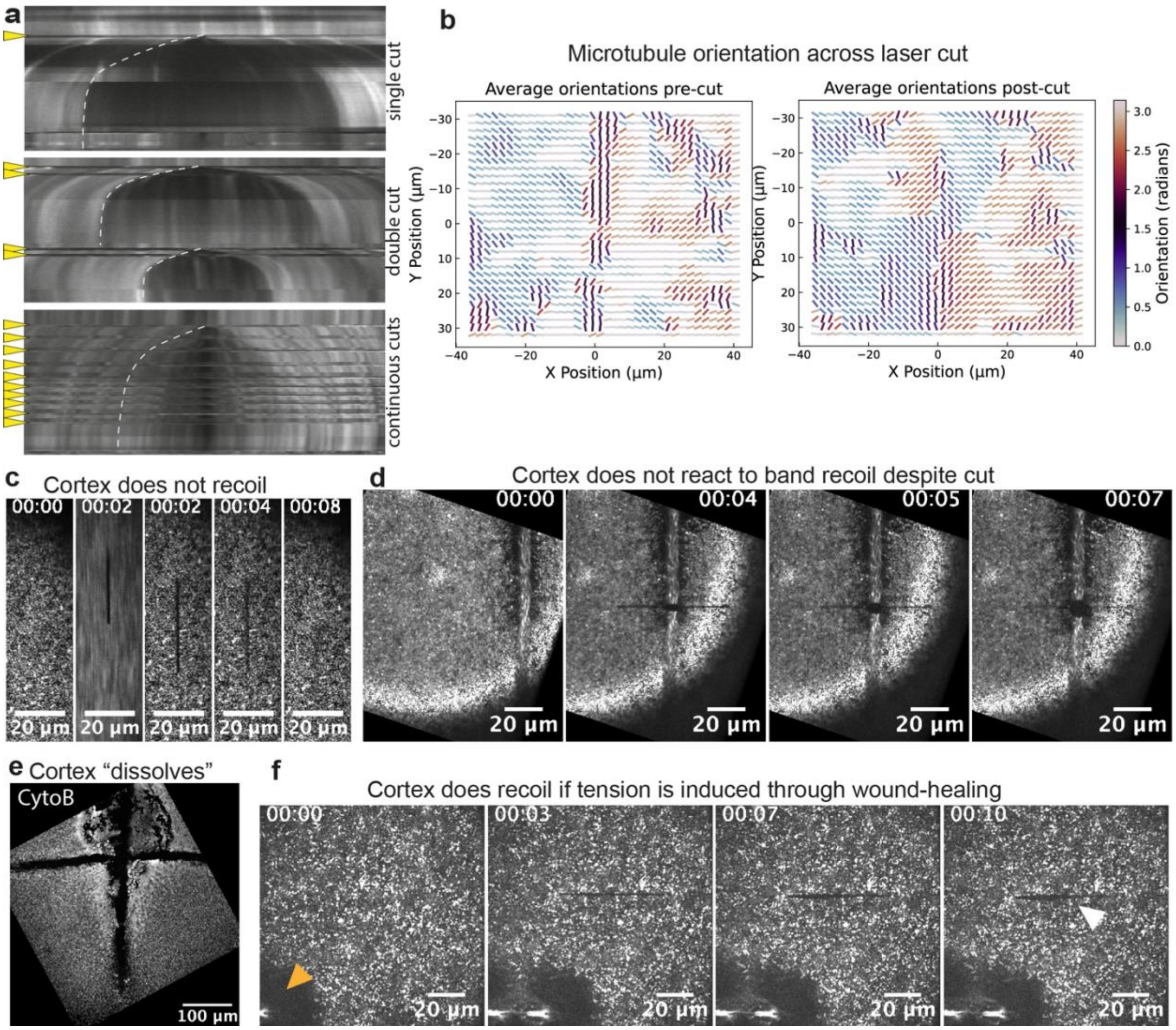

Figure S2

a, Kymographs showing recoil progression after laser ablation of the contractile band, after one cut (top), two times two cuts (middle) and over the course of 10 subsequent cuts. **b,** Microtubule orientations before (left) and 6 seconds after (right) laser ablation, averaged across N=3 individual embryos, automatically detected in Fiji OrientationJ, colour-coded according to angle. The fast imaging causes a detection of the actin band in the microtubule channel (see **Fig 2a**), which is detected as vertical orientation, visible in the pre-cut plot. **c,** Laser cut in the actin cortex, away from the band. Scale bar, 20 µm. **d,** Laser ablation across the contractile band and adjacent cortex. Scale bar, 20 µm. **e,** Maximum intensity projection of zebrafish embryo treated with 10 µg/ml cytochalasin B after the second cell cycle, labelled for actin (utrophin). The contractile bands would usually form where the actin cortex is interrupted. Scale bar, 100 µm. **f,** Laser cut in the cortex, away from the band (white arrowhead), after contractility was induced via wound-healing response (orange arrowhead). Scale bar, 20 µm.

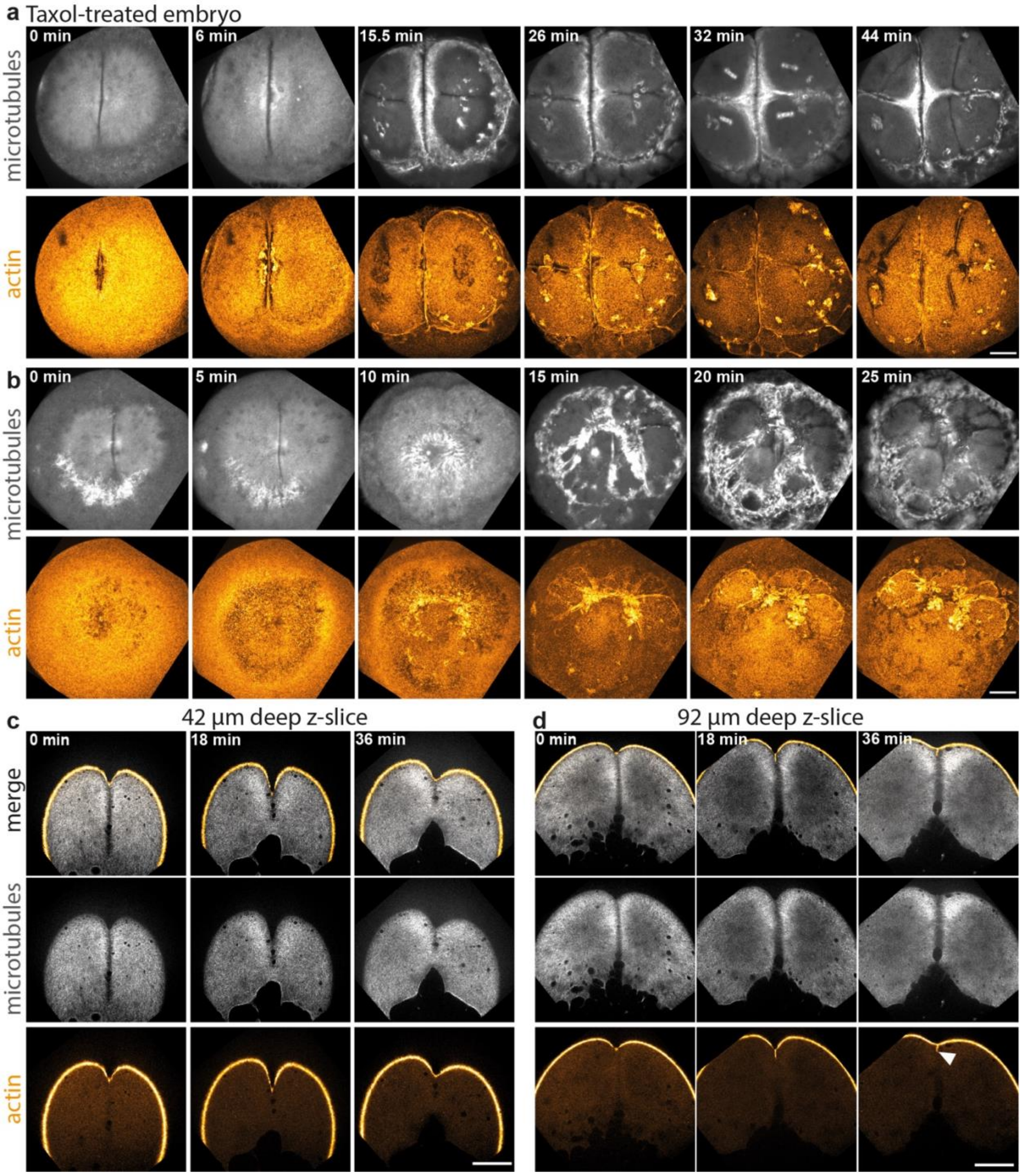
a Taxol-treated embryo
0 min
6 min
15.5 min
26 min
32 min
44 min
microtubules
actin
b
0 min
5 min
10 min
15 min
20 min
25 min
microtubules
actin
c 42 µm deep z-slice
0 min
18 min
36 min
merge
microtubules
actin
d 92 µm deep z-slice
0 min
18 min
36 min

Figure S3

a, Embryo treated with Taxol (0.035 μg/ml in media) during the first three cell cycles of development. The contractile band (from the first division) forms and remains in place throughout the three cycles. **b,** Embryo treated with Taxol (0.07 μg/ ml in media) during the first two cell cycles. The microtubules still show signs of assembly and disassembly during the cell cycle, but the stabilisation of microtubules prevents the contractile band from forming properly. **c,** Embryo treated with cycloheximide to arrest in interphase. Two individual z-planes are shown (corresponding to the maximum intensity projection shown in **Fig 3a**). The furrow ingression after 36 minutes is minimal (white arrowhead). All scale bars, 100 μm.

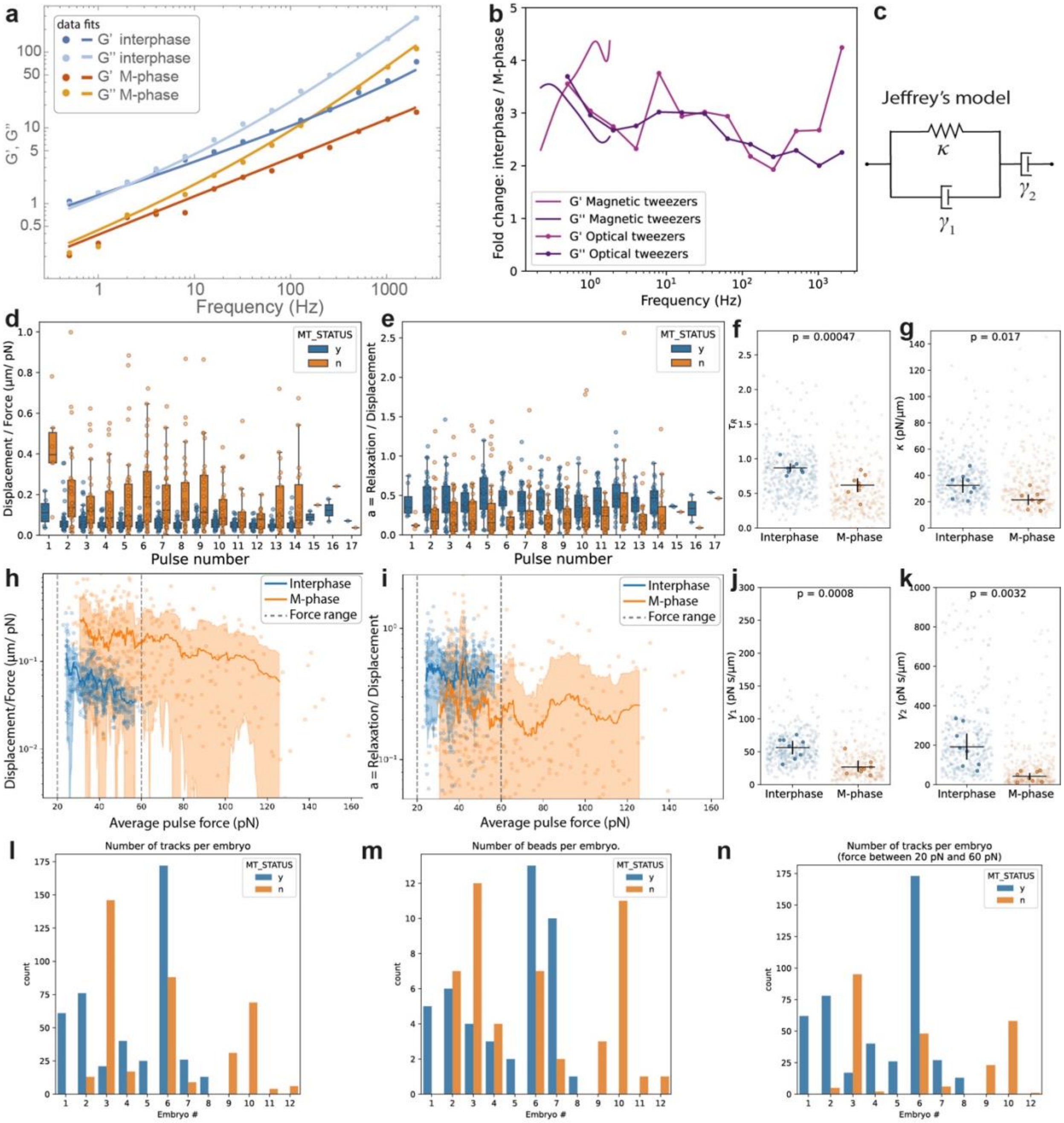
a
data fits
G' interphase
G'' interphase
G' M-phase
G'' M-phase
G', G''
Frequency (Hz)
b
Fold change: interphase / M-phase
G' Magnetic tweezers
G'' Magnetic tweezers
G' Optical tweezers
G'' Optical tweezers
Frequency (Hz)
c
Jeffrey's model
κ
γ1
γ2
d
Displacement / Force (μm/ pN)
MT_STATUS
y
n
Pulse number
e
a = Relaxation / Displacement
MT_STATUS
y
n
Pulse number
f
p = 0.00047
τR
Interphase
M-phase
g
p = 0.017
κ (pN/μm)
Interphase
M-phase
h
Displacement/Force (μm/ pN)
Interphase
M-phase
Force range
Average pulse force (pN)
i
a = Relaxation/ Displacement
Interphase
M-phase
Force range
Average pulse force (pN)
j
p = 0.0008
γ1 (pN s/μm)
Interphase
M-phase
k
p = 0.0032
γ2 (pN s/μm)
Interphase
M-phase
l
Number of tracks per embryo
MT_STATUS
y
n
count
Embryo #
m
Number of beads per embryo.
MT_STATUS
y
n
count
Embryo #
n
Number of tracks per embryo
(force between 20 pN and 60 pN)
MT_STATUS
y
n
count
Embryo #

Figure S4

a, Optical tweezers analysis showing G' and G'' in Interphase (blues), N=5 embryos, n=12 measurements, and M-phase (oranges), N=6 embryos, n=12 measurements. **b,** Ratio of interphase to M-phase for G' and G'' values from optical tweezers measurements (n=12 measurements, respectively) and magnetic tweezers (N=8 individual embryos, respectively) measurements. The mean fold change in optical tweezers measurements for G' is 3.18646 (95% confidence intervals: 3.03078, 3.34214) and for G'' is 3.03712 (95% confidence intervals: 2.89593, 3.17831). **c,** schematic representation of Jeffrey's model. **d,** displacement/ force values for individual embryos in interphase ('y') and M-phase ('n'), N=8 embryos, respectively. Centre line shows median, box shows interquartile range (25th percentile to 75th percentile), whiskers extend to 1.5x IQR, outliers are data beyond whiskers. **e,** relaxation/displacement values for individual embryos in interphase ('y') and M-phase ('n'), N=8 embryos, respectively. Boxes defined as in d. **f,** Results from magnetic tweezers data analysing by fitting to Jeffrey's model. Relaxation time τ, indicating when the beads have relaxed 2/3, N=8 embryos, respectively. p=0.00047, weighted two-sided Student's t-test with unequal variance. Lines show mean and 95% confidence intervals from bootstrapping (1000 resamples), in all Jeffrey's fit data (subfigs f,g,j,k). **g,** κ (pN/µm) from Jeffrey's fit, for individual embryos (transparent) and mean (solid) during interphase (blue) and M-phase (orange), N=8 embryos, respectively. p=0.017 (weighted two-sided Student's t-test with unequal variance). **h,** displacement/force values shown across entire force range. Dashed lines indicate range that was used to compare the two phases, N=8 embryos per cell cycle phase, solid lines show moving average, shaded area shows standard deviation. **i,** relaxation/displacement values shown across entire force range. Dashed lines indicate range that was used to compare the two phases, solid lines show moving average, shaded area shows standard deviation. N=8 embryos, respectively. **j,** γ_1 (pN s/µm) from Jeffrey's fit, after displacement for individual embryos (left) and mean (right) during interphase (blue) and M-phase (orange), N=8 embryos respectively. p=0.0008 (weighted two-sided Student's t-test with unequal variance). **k,** γ_2 (pN s/µm) from Jeffrey's fit, after displacement for individual embryos (left) and mean (right) during interphase (blue) and M-phase (orange), N=8 embryos, respectively. p=0.0032 (weighted two-sided Student's t-test with unequal variance). **l,** number of bead tracks per embryo in interphase ('y') and M-phase ('n'), N=8 embryos, respectively. **m,** number of beads per embryo in interphase ('y') and M-phase ('n'), N=8 embryos, respectively. **n,** number of beads per embryo in interphase ('y') and M-phase ('n'), N=8 embryos, respectively in the used force range of 20-60 pN.

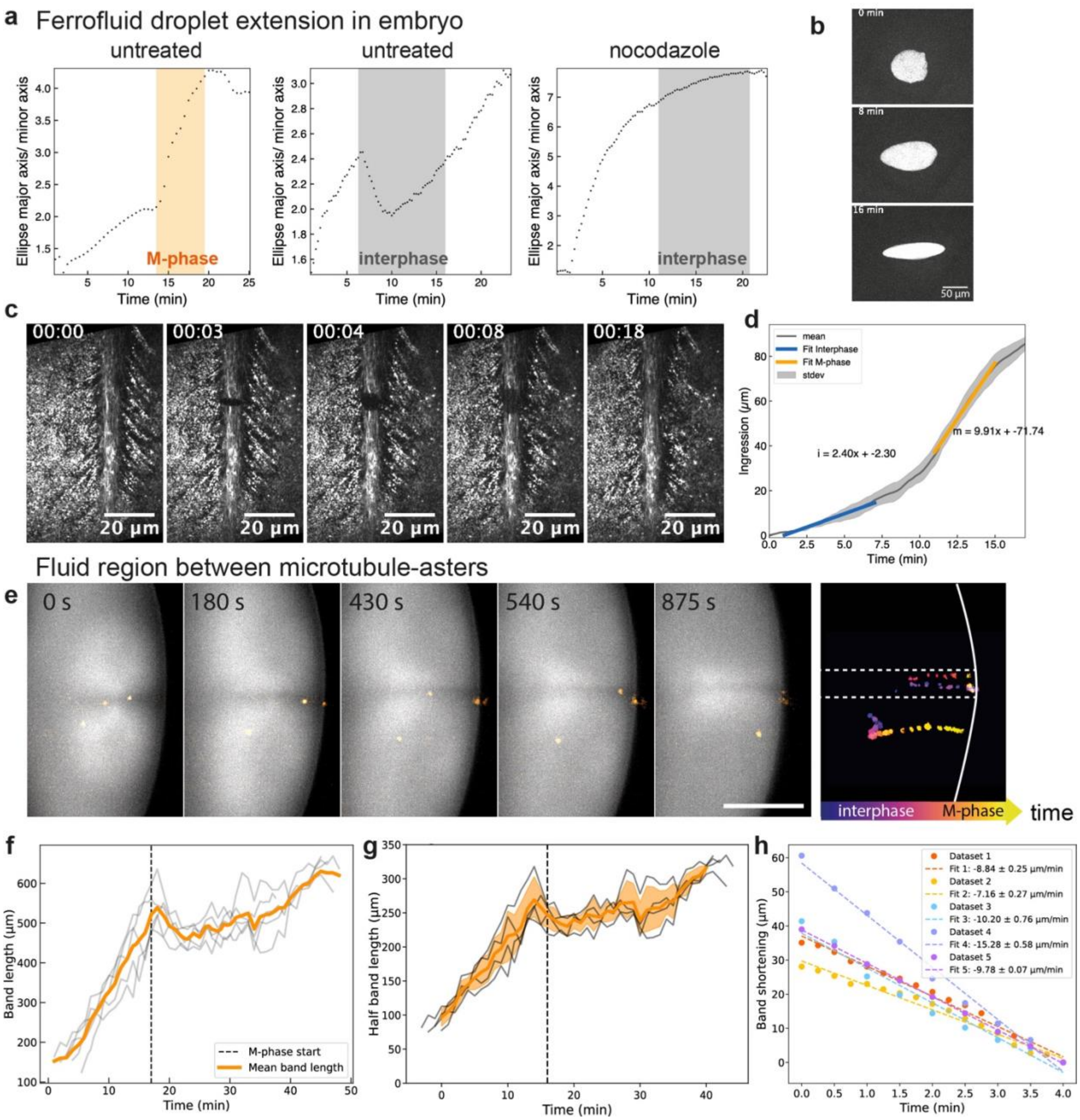

a Ferrofluid droplet extension in embryo
untreated
untreated
nocodazole
Ellipse major axis/ minor axis
Time (min)
M-phase
interphase
interphase
b
0 min
8 min
16 min
50 µm
c
00:00
00:03
00:04
00:08
00:18
20 µm
d
mean
Fit Interphase
Fit M-phase
stdev
Ingression (µm)
i = 2.40x + -2.30
m = 9.91x + -71.74
Time (min)
Fluid region between microtubule-asters
e
0 s
180 s
430 s
540 s
875 s
interphase
M-phase
time
f
Band length (µm)
M-phase start
Mean band length
Time (min)
g
Half band length (µm)
Time (min)
h
Band shortening (µm)
Dataset 1
Fit 1: -8.84 ± 0.25 µm/min
Dataset 2
Fit 2: -7.16 ± 0.27 µm/min
Dataset 3
Fit 3: -10.20 ± 0.76 µm/min
Dataset 4
Fit 4: -15.28 ± 0.58 µm/min
Dataset 5
Fit 5: -9.78 ± 0.07 µm/min
Time (min)

Figure S5

a, Ferrofluid droplet experiment in cytoplasm of untreated and nocodazole treated embryos. Magnet was constantly applied, the bead major axis/ minor axis ratio is plotted, N=1 per phase/condition. **b,** Representative images of ferrofluid in cytoplasm. Scale bar, 50 µm. **c,** Contractile band and actin cortex (labelled by utrophin) over time, visualising wrinkles and response to laser ablation. Scale bar, 20 µm. **d,** contractile band ingression over time in N=4 agarose-confined (mounted) individual embryos, where membrane attachment occurs sooner than in unconfined embryos. Solid line shows mean, shaded area shows standard deviation. **e,** Magnetic tweezer experiment comparing bead behaviour within asters to bead behaviour between asters. Magnetic tip is located to the right of the sample. Right: time projection of bead movement: blue-pink represents interphase, orange-yellow represents M-phase. Scale bar, 100 µm. **f,** Quantification of band length over 45 minutes after band formation onset, measured in N=5 individual embryos (entire band measured in 1 embryo, half of band measured in 4 embryos and multiplied by two for comparison to full band). The data is aligned for the start of M-phase (dashed line). The second interphase starts around 29 min. Orange line shows mean, grey lines show individual measurements (multiplied by two for half band measurement). **g,** bootstrapped data of the band length measurement of N=4 embryos where half of the band (from centre to tip) was measured. Solid orange line shows bootstrapped mean, black lines show individual data, shaded area shows 95% confidence interval of the mean. **h,** band retraction during M-phase and individual fits. Average Slope: -10.25 µm/min, Combined Error (RMSE): 0.20 µm/min, N=5 embryos (plotted as individual lines).

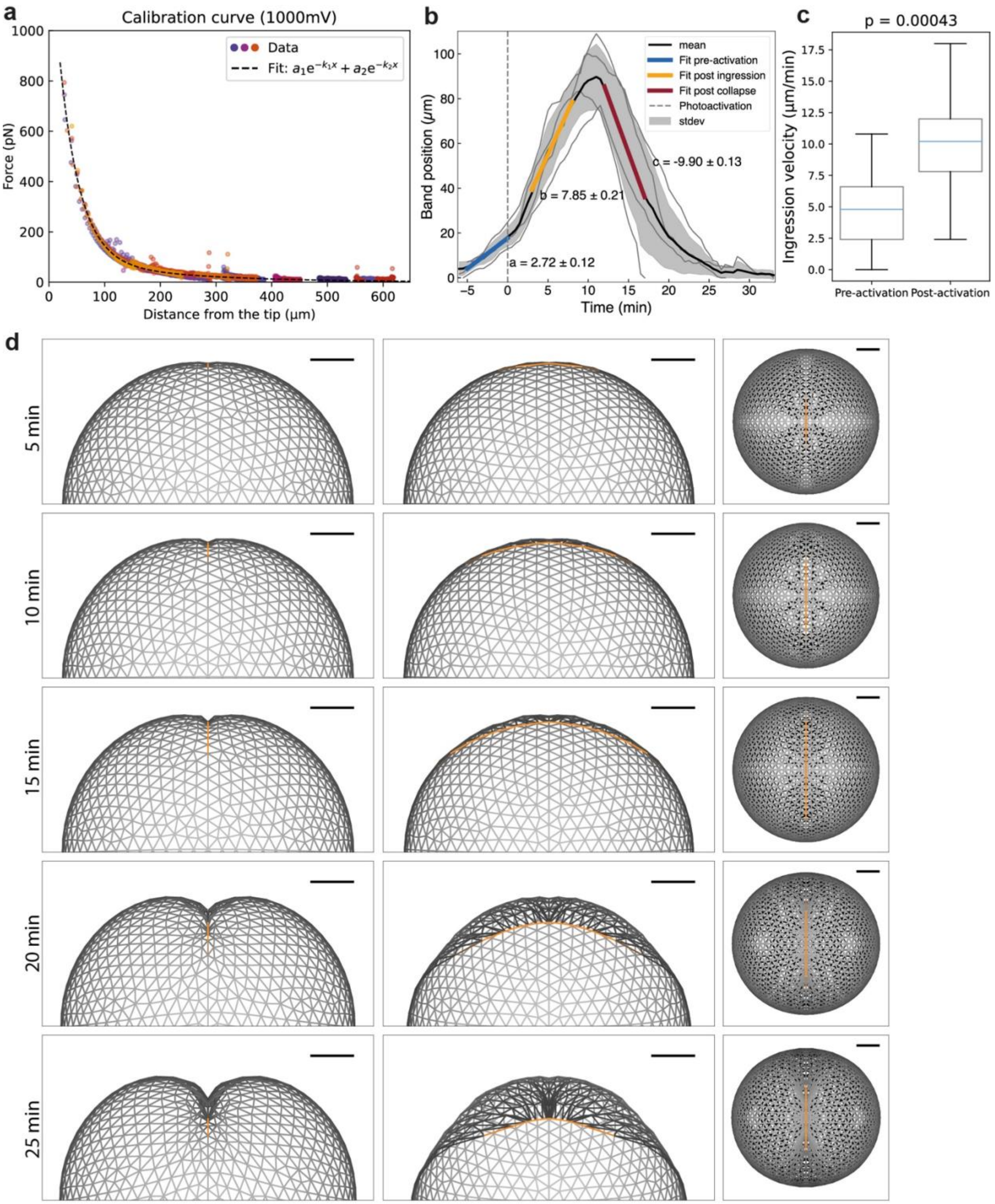
a
Calibration curve (1000mV)
Data
Fit: $a_1 e^{-k_1 x} + a_2 e^{-k_2 x}$
Force (pN)
Distance from the tip (µm)
b
mean
Fit pre-activation
Fit post ingression
Fit post collapse
Photoactivation
stdev
Band position (µm)
Time (min)
a = 2.72 ± 0.12
b = 7.85 ± 0.21
c = -9.90 ± 0.13
c
p = 0.00043
Ingression velocity (µm/min)
Pre-activation
Post-activation
d
5 min
10 min
15 min
20 min
25 min

Figure S6

a, Calibration curve of force (pN) in relation to distance from magnetic tip (µm) for magnetic tweezer experiments. **b,** Ingression trajectory of the contractile band in interphase arrested embryos, before inhibitor-photoactivation (blue, a = 2.72 µm/min), after photoactivation (orange, b = 7.85 µm/min, during ingression "M-phase-like"), and after photoactivation (red, c = -9.90 µm/min) when the ingression was reversed (entire trajectory measured in N=4 individual embryos out of the 17 shown in main figure, entire trajectory measured for visualisation, samples selected based on traceability of the ingression front). Black line shows mean, grey shaded area shows standard deviation. **c,** Ingression velocity of the contractile band in interphase arrested embryos, before (5.143 µm/min) and after (10.255 µm/min) photoactivation of the microtubule inhibitor SbTubA4P in N=4 different embryos (p = 0.00043, paired-sample two-sided t-test). Centre line shows median, box shows interquartile range (25th percentile to 75th percentile), whiskers extend to 1.5x IQR, outliers are data beyond whiskers. **d,** Visualisation of contractile band ingression simulation from front (left), side (centre), and top (right) during interphase (rows 1-3) and M-phase (rows 4 and 5).

Supplementary tables

Supplementary table 1: transgenic zebrafish lines.

Strain	Source	Identifier	Description
Tg(actb2:EGFP-Hsa.DCX)	Norden Lab	cbg5Tg	EGFP-DCX, used for magnetic tweezer experiments
Tg2(actb2:mCherry-Hsa.UTRN)	Norden/ Heisenberg Labs	e119Tg	utrophin-mCherry used for double transgenic line
Tg(actb2:EGFP-Hsa.DCX, actb2:mCherry-Hsa.UTRN)	double transgenic line from above lines		EGFP-DCX; utrophin-mCherry, double transgenic line, used for unperturbed, CHX, CytoB, SbTubA4P, Laser ablation, and obstacle experiments

Supplementary table 2: Microrheology measurement parameters.

Bead size (µm)	Direction (0=y, 1=x)	Sample type (0=liquid, 1=solid)	Ki force clamp integral constant
2.150000	0.000000	0.000000	0.000000
Frequency (Hz)	Amplitude (µm)	te (s)	tm (s)
0.500000	0.100000	20.000000	16.000000
1.000000	0.100000	10.000000	8.000000
2.000000	0.100000	5.000000	4.000000
4.000000	0.100000	3.000000	2.000000
8.000000	0.100000	2.000000	1.000000
16.000000	0.100000	2.000000	1.000000
32.000000	0.100000	2.000000	1.000000
64.000000	0.100000	2.000000	1.000000
128.000000	0.100000	2.000000	1.000000
256.000000	0.100000	2.000000	1.000000
512.000000	0.100000	2.000000	1.000000
1028.000000	0.100000	2.000000	1.000000
2048.000000	0.100000	2.000000	1.000000
4096.000000	0.100000	2.000000	1.000000
6250.000000	0.100000	2.000000	1.000000
