# Supplementary material for: A mechanical ratchet drives unilateral cytokinesis
Source: Nature. 2026 Jan 7;650(8102):759–67. doi: 10.1038/s41586-025-09915-x (PMC12916326; doi:10.1038/s41586-025-09915-x)
Supplement: Supplementary file 2 — Reporting Summary [file 41586_2025_9915_MOESM2_ESM.pdf]

nature portfolio

Corresponding author(s): Jan Brugues

Last updated by author(s): Sep 12, 2025

Reporting Summary

Nature Portfolio wishes to improve the reproducibility of the work that we publish. This form provides structure for consistency and transparency in reporting. For further information on Nature Portfolio policies, see our Editorial Policies and the Editorial Policy Checklist.

Statistics

For all statistical analyses, confirm that the following items are present in the figure legend, table legend, main text, or Methods section.

n/a	Confirmed	
☐	☒	The exact sample size (n) for each experimental group/condition, given as a discrete number and unit of measurement
☐	☒	A statement on whether measurements were taken from distinct samples or whether the same sample was measured repeatedly
☐	☒	The statistical test(s) used AND whether they are one- or two-sided<br>*Only common tests should be described solely by name; describe more complex techniques in the Methods section.*
☒	☐	A description of all covariates tested
☒	☐	A description of any assumptions or corrections, such as tests of normality and adjustment for multiple comparisons
☐	☒	A full description of the statistical parameters including central tendency (e.g. means) or other basic estimates (e.g. regression coefficient) AND variation (e.g. standard deviation) or associated estimates of uncertainty (e.g. confidence intervals)
☐	☒	For null hypothesis testing, the test statistic (e.g. F, t, r) with confidence intervals, effect sizes, degrees of freedom and P value noted<br>*Give P values as exact values whenever suitable.*
☒	☐	For Bayesian analysis, information on the choice of priors and Markov chain Monte Carlo settings
☒	☐	For hierarchical and complex designs, identification of the appropriate level for tests and full reporting of outcomes
☒	☐	Estimates of effect sizes (e.g. Cohen's d, Pearson's r), indicating how they were calculated

Our web collection on statistics for biologists contains articles on many of the points above.

Software and code

Policy information about availability of computer code

Data collection	Data for magnetic tweezers was collected with custom code written in vs code, available under the following link: https://github.com/ursaursic/magnetic_tweezers_brugueslab.
Data analysis	We have included a code availability statement where the entire code is accessible.

For manuscripts utilizing custom algorithms or software that are central to the research but not yet described in published literature, software must be made available to editors and reviewers. We strongly encourage code deposition in a community repository (e.g. GitHub). See the Nature Portfolio guidelines for submitting code & software for further information.

Data

Policy information about availability of data

All manuscripts must include a data availability statement. This statement should provide the following information, where applicable:

- Accession codes, unique identifiers, or web links for publicly available datasets
- A description of any restrictions on data availability
- For clinical datasets or third party data, please ensure that the statement adheres to our policy

We have included a data availability statement, with a doi to access all data.

Research involving human participants, their data, or biological material

Policy information about studies with human participants or human data. See also policy information about sex, gender (identity/presentation), and sexual orientation and race, ethnicity and racism.

Reporting on sex and gender	na
Reporting on race, ethnicity, or other socially relevant groupings	na
Population characteristics	na
Recruitment	na
Ethics oversight	na

Note that full information on the approval of the study protocol must also be provided in the manuscript.

Field-specific reporting

Please select the one below that is the best fit for your research. If you are not sure, read the appropriate sections before making your selection.

☒ Life sciences ☐ Behavioural & social sciences ☐ Ecological, evolutionary & environmental sciences

For a reference copy of the document with all sections, see nature.com/documents/nr-reporting-summary-flat.pdf

Life sciences study design

All studies must disclose on these points even when the disclosure is negative.

Sample size	The sample size was chosen in order to ensure that we have enough samples to be able to rule out that observations were random. This allowed us to perform a statistical test (t-test) in the case of band retraction in different treatments and tension on the band.
Data exclusions	no data exclusion
Replication	Experiments were repeated in multiple embryos from different parents and on different days to ensure reproducibility. In addition, mechanical measurements were performed with two different methods and indicated the same trend.
Randomization	Randomisation was not relevant for this study.
Blinding	The tracking of the beads and detection whether the cell cycle was interphase or M-phase, and the plotting of the tracked data, were done by two individual people to reduce bias.

Reporting for specific materials, systems and methods

We require information from authors about some types of materials, experimental systems and methods used in many studies. Here, indicate whether each material, system or method listed is relevant to your study. If you are not sure if a list item applies to your research, read the appropriate section before selecting a response.

Materials & experimental systems

n/a	Involved in the study	
☐	☒	Antibodies
☒	☐	Eukaryotic cell lines
☒	☐	Palaeontology and archaeology
☐	☒	Animals and other organisms
☒	☐	Clinical data
☒	☐	Dual use research of concern
☒	☐	Plants

Methods

n/a	Involved in the study	
☒	☐	ChIP-seq
☒	☐	Flow cytometry
☒	☐	MRI-based neuroimaging

Antibodies

Antibodies used	anti-myc (human) antibody received from MPI-CBG antibody facility,labelled with DyLight 550 (Thermo Fisher Scientific 84530), were

Antibodies used	used to visualise magnetic beads.
Validation	Validation was not relevant since we used non-specific antibodies solely for labelling beads.

Animals and other research organisms

Policy information about studies involving animals; ARRIVE guidelines recommended for reporting animal research, and Sex and Gender in Research

Laboratory animals	Zebrafish (Danio rerio) of the following strains: Tg(actb2:EGFP-Hsa.DCX), Tg2(actb2:mCherry- Hsa.UTRN), and Tg(actb2:EGFP-Hsa.DCX, actb2:mCherry-Hsa.UTRN)
Wild animals	study does not involve wild animals
Reporting on sex	the sex of embryos was not considered
Field-collected samples	study does not involve samples collected from field
Ethics oversight	The experiments were approved and licensed by the local animal ethics committee (Landesdirektion Sachsen, Germany; license no. DD24.1-5131/ 394/ 33) and executed in accordance with the European Communities Council Directive 2010/63/EU on the protection of animals used for scientific purposes, as well as the German Animal Welfare Act.

Note that full information on the approval of the study protocol must also be provided in the manuscript.

Plants

Seed stocks	na
Novel plant genotypes	na
Authentication	na
